# Nuclear factor I revealed as family of promoter binding transcription activators

**DOI:** 10.1186/1471-2164-12-181

**Published:** 2011-04-07

**Authors:** Milos Pjanic, Petar Pjanic, Christoph Schmid, Giovanna Ambrosini, Armelle Gaussin, Genta Plasari, Christian Mazza, Philipp Bucher, Nicolas Mermod

**Affiliations:** 1 Institute of Biotechnology, University of Lausanne, and Center for Biotechnology of the University of Lausanne and École Polytechnique Fédérale de Lausanne, CH-1015 Lausanne, Switzerland; 2 Software Examination and Certification Laboratory, Faculty of Mathematics, University of Belgrade, 11000 Belgrade, Serbia; 3 Swiss Institute for Experimental Cancer Research, École Polytechnique Fédérale de Lausanne, CH-1015 Lausanne, Switzerland; 4 Swiss Tropical and Public Health Institute, Socinstrasse 57, P.O. Box, CH-4002 Basel, Switzerland; 5 Universität Basel, Petersplatz 1, CH-4003 Basel, Switzerland; 6 Swiss Institute of Bioinformatics, EPFL SV ISREC, AAB 0 09 (Bâtiment AAB), Station 15, CH-1015 Lausanne, Switzerland; 7 Department of Mathematics, University of Fribourg, CH-1700 Fribourg, Switzerland

## Abstract

**Background:**

Multiplex experimental assays coupled to computational predictions are being increasingly employed for the simultaneous analysis of many specimens at the genome scale, which quickly generates very large amounts of data. However, inferring valuable biological information from the comparisons of very large genomic datasets still represents an enormous challenge.

**Results:**

As a study model, we chose the NFI/CTF family of mammalian transcription factors and we compared the results obtained from a genome-wide study of its binding sites with chromatin structure assays, gene expression microarray data, and in silico binding site predictions. We found that NFI/CTF family members preferentially bind their DNA target sites when they are located around transcription start sites when compared to control datasets generated from the random subsampling of the complete set of NFI binding sites. NFI proteins preferably associate with the upstream regions of genes that are highly expressed and that are enriched in active chromatin modifications such as H3K4me3 and H3K36me3. We postulate that this is a causal association and that NFI proteins mainly act as activators of transcription. This was documented for one member of the family (NFI-C), which revealed as a more potent gene activator than repressor in global gene expression analysis. Interestingly, we also discovered the association of NFI with the tri-methylation of lysine 9 of histone H3, a chromatin marker previously associated with the protection against silencing of telomeric genes by NFI.

**Conclusion:**

Taken together, we illustrate approaches that can be taken to analyze large genomic data, and provide evidence that NFI family members may act in conjunction with specific chromatin modifications to activate gene expression.

## Background

High-throughput assays are being widely employed in various fields of biology. For example in genomics, DNA microarrays are used to simultaneously measure the expression levels of nearly all genes of a genome [1,2]. Recently, a new high-throughput method has been developed for a whole genome mapping of protein-DNA interactions that is based on the chromatin immunoprecipitation and next generation sequencing technology (method termed chromatin immunoprecipitation sequencing or ChIP-Seq) [3-8]. These two high-throughput methods, when combined, are instrumental to study how transcription factors regulate gene expression at a global genomic scale. As the costs of new-generation sequencing and DNA microarrays decrease, such high-throughput assays should be increasingly used. In addition, new software tools are emerging rapidly, allowing faster and easier analyses of large-scale genomic datasets [9-13]. However, extracting the significant and biologically relevant information from such massive datasets still represents a great challenge. In purely experimental studies, the use of negative controls such as the blank or mock conditions is absolutely necessary. However, genome-wide computer analyses may lack an adequate negative control. In such case, a randomly selected portion of the total dataset can be used as an *in silico *negative control, such as for instance a randomly picked sample of all genomic loci. The size of the control dataset can then be chosen to parallel that of the experimental set, to simplify the statistical analysis [14,15].

As a study group of proteins, we chose NFI/CTF family of mammalian transcription factors. NFI/CTF represents a family of transcription-replication factors comprising polypeptides encoded by four paralogous genes located on different chromosomes in mammals (NFIA, NFIB, NFIC, NFIX) [16,17]. NFI family of proteins displays the unusual property of regulating not only the initiation of transcription but also of mediating DNA replication [18]. NFI recognition sequence were found in the promoter sequences of many cellular genes [19], where they may act as activator or repressor of transcription [20-25]. Recently, it has been proposed that NFI may be involved in a long range regulation of gene expression, through the formation of chromatin barrier and by blocking the propagation of a heterochromatic structure [26,27]. NFI binds as a dimer, and its preferred binding sequence is a palindrome composed of two half sites TTGGCANNNTGCCAA. A position weight matrix for the NFI/CTF was established using a collection of over 10,000 SELEX-SAGE selected sites, allowing the prediction of its binding affinity to any genomic sequence [28]. However, since this prediction matrix is based on NFI binding specificity *in vitro*, the specificity of this family of proteins may be different from that observed in the cell, where interactions with other transcription factors may take place and DNA accessibility may be restrained by chromatin. Here we assessed the in vivo binding preferences of NFI/CTF, its global functional properties regarding the regulation of gene expression and the relationship of NFI binding sites with different histone methylation markers typical of either an open or closed chromatin structure.

## Results

### NFI preferentially binds upstream of transcription initiation sites in mouse genome

Statistical analysis in genomics often relies on the sub-sampling of datasets, which requires random sampling algorithms. We devised a random sampling algorithm that can be conveniently applied to large genomic datasets. The random sampling algorithm C++ source code is available as a text file online (Additional file 1). Each randomly generated number is used to extract an entry line from the main dataset to generate a subset of the desired size. Simulation experiments indicated that subsampling can be applied to sets of normally distributed values without loosing the statistical robustness of the comparisons, provided that relatively large subsets of data are retained (e.g. equal or greater than 100 individual values; see Additional file 2).

We first used this random sampling method to compare data from a ChIP-Seq experiment performed on primary mouse embryonic fibroblasts for the NFI transcription factor family relative to the in silico predictions of its binding sites. The mouse genome (NCBI build 37 or mm9) was found to contain a set of 61,492 NFI predicted sites that were defined using a previously established position weight matrix [19,28]. The predicted sites were defined with a matrix score threshold > 85, which corresponds to a medium *in vitro *binding affinity in the range of scores that extends from a minimum of -108 to a maximum of 100. Within this set, 2,852 predicted sites overlapped DNA sequences covering the RefSeq annotated transcription start sites (TSS) and 5 kb of upstream sequences.

**Figure 1:**
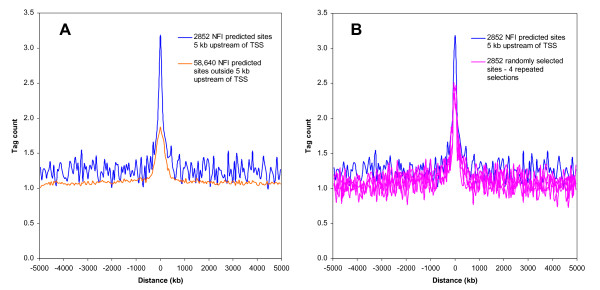
**NFI family of DNA binding proteins preferentially binds upstream of initiation sites of transcription in mouse genome**. A set of 61,492 NFI predicted sites was defined in the mouse genome, NCBI build 37 or mm9. Sites were defined with a previously established position weight matrix (sequence score threshold > 85, out of maximum 100) [28]. Within this set, 2,852 predicted sites overlapped regions 5 kb upstream from RefSeq annotated transcriptional start sites (TSS). As a negative control for this experiment, a complementary dataset of 58,640 sites was selected (panel A) or the same number of NFI predicted sites (2,852) were randomly selected from the initial set of 61,492 predicted sites (panel B). Four independently performed random selection are shown. Average NFI ChIP-Seq tag counts were calculated in windows of 50 bp for a region of 5 kb up- and down-stream of the selected NFI predicted sites. Tag counts were normalized globally, as a fold increase over the genome average tag count in a window of 50 bp. Obtained data points were connected to form a continuous line.

Actual *in vivo *binding site occupancy was estimated using the average ChIP-Seq tag count covering each subset of NFI predicted sites. When tag counts occurring at TSS-proximal binding sites were compared to those that do not occur near known TSS, a higher tag counts was noted at the predicted sites, but also in regions extending several kb away from the known binding sites (Figure 1A). Since the DNA fragments used in this assay did not cover a size range extending over several kb, we reasoned that this might either result from a surprisingly frequent association of the protein to sequences near binding sites, or, alternatively, that it may stem from the smaller size of the dataset comprising the TSS-proximal 2,852 binding sites relative to the non-TSS datasets of 58,640 sequences, resulting in a relatively noisier distribution at non-bound sequences, or from some other artefactual effects associated to the method.

To assess the latter possibility further, we generated control datasets of the same size as the experimental set by randomly selecting 2,852 sequences from the 61,492 predicted sites. Comparison of distinct randomly selected data subsets indicated comparable tag counts at predicted binding sites, and similar signal-to-noise ratio. Thus, the sampling method provided a reliable estimation of the binding site occupancy, since several random groups of predicted sites did not differ markedly in their protein occupancy (Figure 1B). As before, the dataset corresponding to TSS-proximal sites showed a more prominent tag count around the predicted sites. However, comparable tag counts were observed within 500 to 5000 bp windows around the predicted binding sites, when comparing the experimental profiles to those of control datasets of the same size. Thus, we conclude that the group of TSS-proximal predicted sites displayed higher protein occupancy at predicted sites when compared to the randomly selected binding sites, but that it was not the overall promoter region that was more frequently bound by the protein. This suggested that binding sites within genomic loci upstream of core promoters may bind NFI with higher apparent affinity.

### NFI-bound genes show higher expression levels

**Figure 2:**
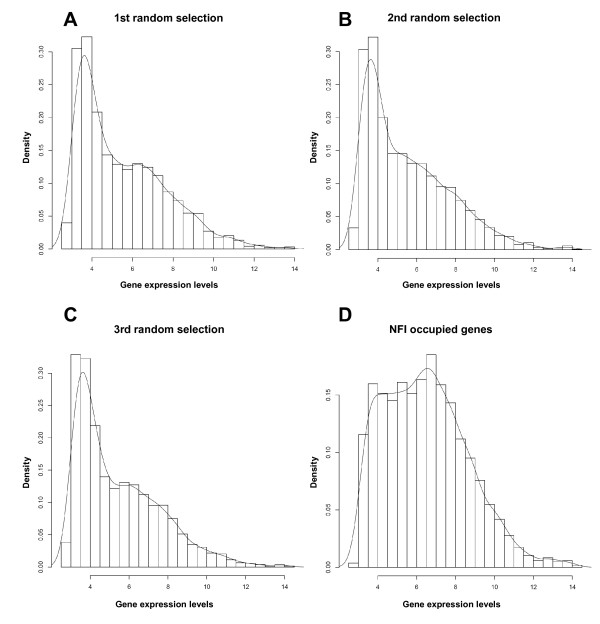
**Genes that contain NFI *in vivo *site in their 5 kb upstream regions exhibit higher expression levels**. Using ChIP-Seq data, 39,807 *in vivo *NFI sites were defined genome-wide in mouse embryonic fibroblasts. Out of this number 3,120 *in vivo *sites were located within the 5 kb upstream regions of RefSeq annotated genes. 2881 RefSeq genes were selected that contained one or more *in vivo *NFI sites in their 5 kb upstream regions and the same number of genes were randomly chosen from the RefSeq gene annotation. Random selection was repeated 3 times. Histograms represent the distribution of gene expression levels for each of such defined groups. Affymetrix expression data were obtained from the same cell type (mouse embryonic fibroblasts), from the same embryo, and using the same culturing conditions. A-C. Randomly selected groups. D. Group of NFI occupied genes.

Next, we assessed whether genes that bind NFI may have some common features regarding their gene expression levels. We selected 39,807 *in vivo *NFI sites genome-wide from the ChIP-Seq data, which corresponds to the genomic loci obtained from the collection of precipitated DNA fragments. Out of this number, 3,120 *in vivo *sites were located within 5 kb upstream regions of RefSeq annotated genes. As multiple *in vivo *NFI sites may occur within the 5 kb upstream regions, a total of 2881 RefSeq-annotated genes were identified to contain one or more *in vivo *NFI sites. Control groups were generated to consist of 2,881 genes randomly selected from the total database of RefSeq genes. The expression levels of these genes was assessed using microarray profiling data of mRNAs obtained from murine embryo fibroblasts [29]. Again, randomly selected groups of genes did not differ significantly in their overall expression levels or in their distribution profiles, with the most prominent peak corresponding to lowly expressed genes (Figure 2A-C). However, the transcriptional levels of the group of NFI-containing genes were distinctly higher than those of the control subgroups (Figure 2D), with a median expression value of 6.34 for NFI-bound genes versus 4.93, 4.97 and 4.82 for the randomly selected groups of genes (two tailed t-test: p = 8.94 × 10^-61^, 1.15 × 10^-57^, 1.35 × 10^-65^, respectively). Thus, we conclude that NFI preferentially occupies promoters or upstream regulatory regions of genes that exhibit high expression levels in the cell.

### NFI binding correlates with specific histone methylation patterns

**Figure 3:**
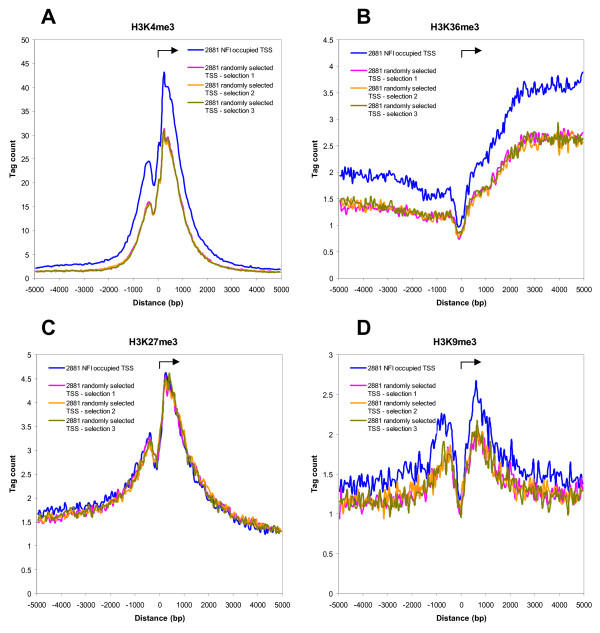
**NFI binding correlates with histone H3 methylations: H3K4me3, H3K36me3, H3K9me3**. 2881 NFI occupied genes in mouse embryonic fibroblasts and 3 groups of randomly selected genes were defined as in Figure 2. ChIP-Seq data for 4 different histone H3 methylations were obtained from the same cell type (mouse embryonic fibroblasts) [4]. Average ChIP-Seq tag counts were calculated in windows of 50 bp for a region of 5 kb up- and down-stream of the orientated transcription start sites (TSS). Tag counts were normalized globally, as a fold increase over the genome average tag count in a window of 50 bp for the following modifications: A. H3K4me3, B. H3K36me3, C. H3K27me3, and D. H3K9me3. Obtained data points were connected to form a continuous line. Arrows indicate the orientation of transcription in each panel.

To test whether the preferential binding of NFI to upstream regions of expressed genes may be associated with specific chromatin modifications, we used ChIP-Seq data for 4 different histone H3 methylations, as previously obtained from mouse embryonic fibroblasts [4]. We created average ChIP-Seq tag profiles surrounding the TSS for each of these modifications. The group of 2,881 NFI bound genes showed higher levels of H3K4me3 and H3K36me3, which are markers of active promoters and transcribed regions, respectively, when compared to the three groups of 2,881 randomly chosen genes (Figure 3A, B). Interestingly, NFI binding at TSS did not correlate with a modification often associated with non-expressed or bivalent chromatin, H3K27me3, when compared with the profiles of the control gene subgroups (Figure 3C), indicating that NFI binding does not occur at promoter regions that are generally enriched in all histone modifications. Interestingly, we found H3K9me3, a modification recently associated with NFI chromatin-domain boundary activity at telomeres [26], to be also enriched surrounding the TSS of the group of NFI-bound genes (Figure 3D).

### NFI-C most often acts as an activator of gene expression

**Figure 4:**
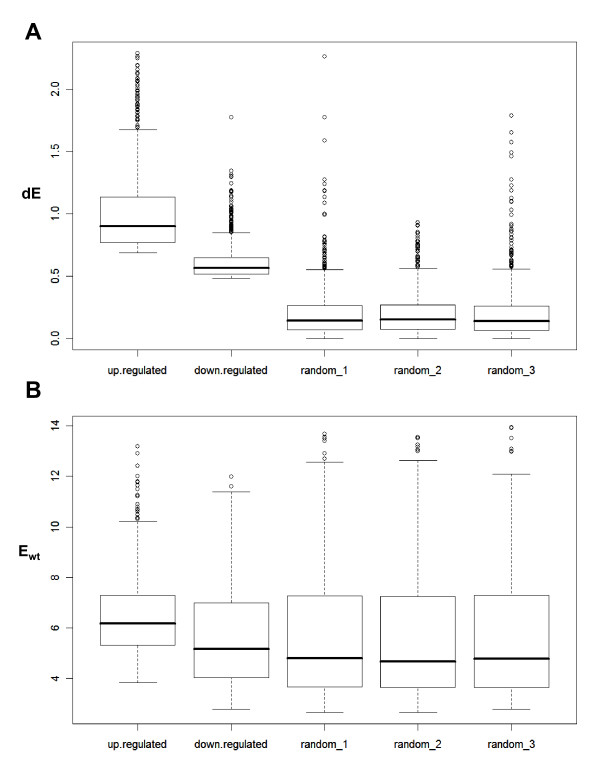
**NFI-C transcription factor acts as more potent activator of gene expression than repressor**. A. Differences in the gene expression levels in wild-type (WT) and NFI-C knock-out (KO) mouse embryonic fibroblasts were depicted for 1000 most up-regulated genes and 1000 most down-regulated genes by NFI-C, as well as for 3 independently selected random groups of 1000 genes. On the y-axis: for each gene in such defined groups expression level in knock-out cells was subtracted from the WT expression level (E_wt_-E_ko_) and plotted as an absolute value. B. Affymetrix expression levels in WT cells (E_wt_) for 1000 most up-regulated genes by NFI-C, 1000 most down-regulated genes by NFI-C, and 3 sets of 1000 randomly chosen genes.

The preferential association of NFI with expressed genes prompted us to test whether NFI family members may have the potential to activate gene expression, or whether their binding is rather the consequence of histone modifications and chromatin accessibility at promoters. For this purpose, we used gene expression microarray data from mouse embryonic fibroblasts in which one of the NFI factors (termed NFI-C) was knocked out by insertional mutagenesis [30]. We focused on differences in expression levels when comparing wild-type (WT) and knock-out cells (KO), and defined a set of 1000 genes which expression was most decreased in the absence of NFI-C (i.e. genes potentially directly activated by this factor). We also defined the set of 1000 genes whose expression is most increased in the absence of NFI-C (i.e. genes potentially directly repressed by NFI-C). We also randomly sampled 1000 genes from all RefSeq genes as control sets. Differences in the microarray signals of WT and KO cells (ΔE = E_wt_-E_ko_) were determined for each gene within defined groups and plotted as an absolute value. The group of genes activated by NFI-C showed the highest median ΔE value, implicating this factor as a potent gene activator (Figure 4A). NFI-C suppressed genes also appeared to be significantly regulated as compared to the control groups. However, NFI-C suppressed genes displayed moderate ΔE values, between those of the control groups and of the group of NFI-C activated genes, indicating that NFI-C is globally a less potent inhibitor of gene expression. When considering the expression levels in WT cells (E_wt_), the group of NFI-C suppressed genes had levels of expression that did not differ significantly from the control subgroups (median expression value: 5.16 for down-regulated genes versus 4.81, 4.67 and 4.78 for the control gene groups, two tailed t-test: p = 0.97, 0.57, and 0.50, respectively), again indicating that NFI-C acts as a moderate inhibitor of gene expression (Figure 4B). Overall, the group of NFI-C activated genes had significantly higher levels of expression than the control groups (median expression value: 6.18 for up-regulated genes versus 4.81, 4.67 and 4.78 for random gene groups; two tailed t-test: p = 2.04 × 10^-19^, 3.22 × 10^-21^, 1.60 × 10^-22^, respectively). These results suggest that NFI-C member of the NFI family acts globally as an effective gene activator.

## Discussion and Conclusions

In this study, we used a random sampling procedure as a general method to obtain reliable control datasets in the analysis of high-throughput genomic assays. We find that datasets of more than 100 individual values can be used without decreasing the robustness of statistical analysis, and that independently generated random subsets of data have statistically indistinguishable global properties. Thus, subsampling can provide a convenient way to display and compare the noise and signals from experimental and control datasets of the same size.

First, we showed that NFI binds preferentially those predicted sites that are located upstream of the initiation sites of transcription (Figure 1). Several interpretations may be given to the preferential association of NFI to binding sites in the proximity of TSS rather than to other locations of the genome. It is known that NFI occupies the promoters of many genes where it may bind synergistically with some other transcription factors such as hepatocyte nuclear factor 1 alpha, estrogen receptor, Brg-associated factor [31-33]. Thus, the preferred occupancy of TSS proximal sites may at least in part reflect the synergistic association of NFI with other factors.

We also found that NFI occupy promoters or upstream regions of the group of genes that are significantly more expressed than the representative randomly selected control groups. Since correlation does not necessarily imply causal relationship, this observation does not allow the conclusion that NFI-family members actually activate the expression of these genes. For instance, NFI might bind highly expressed genes to suppress in part their expression, but still leaving relatively high transcription levels. However, taken together with previous observations that NFI activates the expression of many genes in higher eukaryotes [20,21,32,34-37], we rather conclude that the observed correlation may originate from a direct up-regulation of gene expression by NFI, at least for a significant proportion of its target genes.

The hypothesis that NFI family members may directly activate genes appears to be true for at least one of the member of the family (i.e. NFI-C), as mRNA profiling analysis performed on wild-type and NFI-C knock-out cells revealed that NFI-C is a more potent gene activator than a repressor. The 1000 genes that are most up-regulated by NFI-C had significantly higher change in their expression levels than the top 1000 down-regulated genes. In addition, up-regulated genes showed significantly higher expression levels than representative control gene samples selected from the total gene population, implicating again that this factor is a potent activator of gene expression. Since the selected *in vivo *NFI binding sites are located up to 5 kb from their TSS, which is a relatively large distance, NFI might act as well through some of the types of remote regulation, for instance by the establishment of a chromatin domain boundary that would prevent the propagation of a silencing chromatin structure towards the promoter [27,38].

Histone H3 methylations such as the H3K4me3 and H3K36me3 modifications were found to be enriched around the TSS of NFI-occupied genes when compared with control gene groups. This finding is consistent with the model that NFI acts predominantly as an activator of transcription, since H3K4me3 and H3K36me3, but not H3K27me3, were proposed as markers of active gene transcription [4,6,39]. This indicates that NFI binding to the upstream regions may contribute to the recruitment of the specific enzymes for the H3K4me3 and H3K36me3 modifications. A genome-wide correlation of the occurrence of H3K27me3 was also observed around TSS occurring close to NFI-bound sites, however it was indistinguishable to that of the control group of genes. This indicates that this correlation results from an enrichment of H3K27me3 around at least some of the TSS, and that NFI is not involved in the recruitment of enzymes mediating this modification. Thus, the enrichment of H3K27me3 modification over the NFI bound genes represents a false positive genome-wide correlation. Interestingly, we also found the H3K9me3 modification to be slightly enriched in the group of NFI bound genes. Although H3K9me3 has been associated with a closed chromatin structure, this suggests that NFI may be involved in the recruitment of enzymes that mediate this modification. Interestingly, this modification was recently associated with a chromatin domain boundary effect at telomeric regions in human cells [26]. In this study, NFI was shown to prevent the propagation of a silencing chromatin structure from the telomere, and the expressed genes protected from telomeric silencing by NFI were shown to have elevated H3K9me3 marks at specific telomeric positions. Thus, we may conclude from these studies that the enrichment in H3K9me3 may be a hallmark of gene expression activation by NFI.

## Methods

### Cell culture

Mouse embryonic fibroblasts (MEF) were extracted from mouse embryos of 14.5 days. Cells from WT (wild-type) and NFI-C knock-out embryos were cultured under the following conditions: 37°C, 5% CO_2_, DMEM (GIBCO, 41966), Supplementary 10% FBS (GIBCO, Fetal Bovine Serum, qualified origin US, 26140-079), 1% v/v nonessential amino-acids (GIBCO, 11140-035), 1% v/v L-glutamine (GIBCO, 25030-024).

### Chromatin Immuno-precipitation (ChIP)

Chromatin was extracted from approximately 20,000,000 primary mouse embryonic fibroblasts grown in culture and cross-linked using 11% formaldehyde. Extracted chromatin was fragmented to the average fragment size of 1000 bp using high-frequency sound sonication on VibraCell-75455 (Bioblock Scientific). ChIP was performed as described before [27] using the commercial antibody against NFI group of proteins (NFI (H300): sc-5567, SantaCruz Biotechnology). Antibody complexes were precipitated using rProtein A Sepharose Fast Flow (Amersham Biosciences).

### Illumina/Solexa sequencing

ChIP DNA was processed using the contents of the ChIP-Seq Sample Prep Kit (Illumina). Size range of templates was selected by loading the entire processed sample on a 2% agarose gel and excising the gel region of 50-400 bp. PCR amplification of the gel-extracted DNA was performed for 18 cycles using the adapter-specific primers. Each sample was loaded into 3 separate flow cell channels of the Illumina Cluster Station and then subjected to sequencing-by-synthesis on the Illumina Genome Analyzer sequencing system. For each of the samples, sequence reads from independent channels were pooled together.

### Data analyses

Clustering and correlation analyses of mapped reads were performed using ChIP-peak and ChIP-cor tools available on the ChIP-Seq Analysis Server [40,41]. *In vivo *NFI sites were defined using the ChIP-peak tool and applying the following parameters: window width - 300 bp, vicinity range - 300 bp, peak threshold - 5 tags, count cut-off - 1 tag, repeat filtering-on. Correlation analyses were made using ChIP-cor tool and applying the following parameters: window width - 50 bp, count cut-off - 1, normalization - global. Galaxy tools were used to operate on the genomic intervals [42,43].

### Random sampling algorithm

Random sampling algorithm was written in C++ language and compiled in Microsoft Visual Studio as detailed in the Additional file 1 online. The algorithm uses the computer system date and time as a constantly increasing number for seeding the random number generators at each independent run of the random number generator. Each random number so generated was used to select a single entry line from the dataset to be sub-sampled, with the limitation that a single line of the input file could be selected only once. The source code for random sampling algorithm is available in the additional materials online (Additional file 1).

### Datasets repository

ChIP-Seq data for the wild type and NFI-C knock-out mouse embryonic fibroblasts were deposited at the Gene Expression Omnibus (GEO) repository under the accession number GSE15844. Gene expression microarray data for the wild type and NFI-C knock-out mouse embryonic fibroblasts were taken from the GEO repository under the accession number GSE15871. ChIP-Seq data for histone methylations in mouse embryonic fibroblasts (H3K4me3, H3K9me3, H3K27me3, H3K36me3) were taken from GEO repository under the accession number GSE12241.

## List of Abbreviations

NFI: Nuclear Factor I; NFI-C: Nuclear Factor I - C; CTF: CAAT box transcription factors; ChIP: Chromatin immuno-precipitation; ChIP-Seq: Chromatin immuno-precipitation sequencing; MEFs: Mouse embryonic fibroblasts; TSS: Transcriptional start site; TFs: Transcription factors; NFI-C KO: Nuclear Factor I - C knock out; WT: Wild type

## Authors' contributions

MP conceived the study, carried out the ChIP-Seq experiments, participated in the data analysis and drafted the manuscript. PP developed the random sampling algorithm and participated in the data analysis. CS, GA and PB developed ChIP-Seq analysis tools and participated in the data analysis. AG, GP made substantial contributions to the data collection and interpretation of data. CM, PB and NM participated in the design of the study, made substantial contributions to the interpretation of data, provided essential data revisions, and helped to draft the manuscript. All authors have read and approved the final manuscript.

## Supplementary Material

Additional file 1 (Source code of the random sampling algorithm, text file). Click here for file

Additional file 2 (Construction and statistical properties of random sampling method, supplemental figures relating to the construction and statistical properties of the random sampling method). Click here for file
