# Supplementary material for: Nuclear factor I revealed as family of promoter binding transcription activators
Source: BMC Genomics. 2011 Apr 7;12:181. doi: 10.1186/1471-2164-12-181 (PMC3082249; doi:10.1186/1471-2164-12-181)
Supplement: Additional file 2 — (Construction and statistical properties of random sampling method, supplemental figures relating to the construction and statistical properties of the random sampling method). [file 1471-2164-12-181-S2.PDF]

Supplemental Methods File.

Generation of a random number generator and a random sampling algorithm

Uniform distribution of 1,000,000 random numbers is shown for the whole range in a histogram (Supplemental Figure 1) and dot-chart (Supplemental Figure 2). In addition, the algorithm does not show any gaps in its range. To show this we selected 10,000,000 random numbers and plotted their distribution in the range 0-300, using the bin window of 1. The plot shows that the algorithm does not make any gaps and all the numbers in the range 0-300 could be selected (Supplemental Figure 3).

The source code of the random sampling algorithm was used in this study to generate random numbers that were used in turn to select the lines from the input dataset. The user needs to specify at the entry how many lines are to be chosen by the program. We also introduced a limit that a single line of the input file could be selected only once. After compiling in Microsoft Visual C++, the user should type in the command line: the name of the exe file, followed by first the desired name of the output file and then the name of the input file, e.g. :

name_of_the_program.exe output_file.txt input_file.txt

After calling the exe file in such way, the user will be asked how many entry lines to select from the input file in a random manner.

As a final step, we wished to assess the robustness of comparisons of datasets of different sizes. Datasets of widely different sizes may be heteroscedastic, that is of unequal variance, which was linked to an overestimation of the difference between the datasets in some statistical analysis [1, 2]. However, random sub-sampling of the larger dataset may reduce the power of the test, as the size of the tested dataset becomes smaller. This latter

possibility was assessed using a simulation experiment, where two sets of 10^5 normally distributed numbers were picked randomly to have either identical means of 10, or different means of 10 and 11, using the R software (R Development Core Team, 2006. R: A language and environment for statistical computing. R Foundation for Statistical Computing, Vienna, Austria. IURL http://www.R-project.org, R Software version 2.3.1). The standard deviations were chosen to be the same and equal to 1 in all cases.

Then, the above-described random sampling algorithm was used to sub-sample these groups as performed in genomic analysis, to define subsets of either 10^4, 10^3, 10^2 or 10^1 values. A standard two-tailed t-test was used to compare selected sub-samples and the means and standard deviations of p-values were recorded. In either case of equal or non-equal means, the statistical comparison of datasets of identical sizes yielded similar p values as the comparisons of datasets of different sizes, when the dataset sizes were greater than or equal to 10^2 (Supplemental Figure 4). However, the comparisons of sets of different means yielded erroneous conclusions (p-values >0.05) when at least one dataset of 10^1 values of was used. Thus, random sub-sampling does not decrease the power of the statistical test for datasets of sufficiently large sizes, e.g. of 100 values or more. In addition, random sampling of predicted binding affinities or ChIP-Seq tag occurrence did not change the variance of the subsets in comparison to the overall set of data (data not shown). Thus, we conclude that random sampling of genomic samples in itself will not introduce biases or decrease the robustness of statistically assays, or otherwise cause problems related to heteroscedacity in subsequent parametric statistical analysis.

1. Gamage J, Weerahandi S: **Size performance of some tests in one-way anova**. *Communications in Statistics - Simulation and Computation* 1998, **27**(3):625 - 640.
2. Wittkowski KM: **Statistical analysis of unbalanced and incomplete designs - experiences with BMDP and SAS**. *Statistical software newsletter* 1991.

Supplemental Figures 1-4.

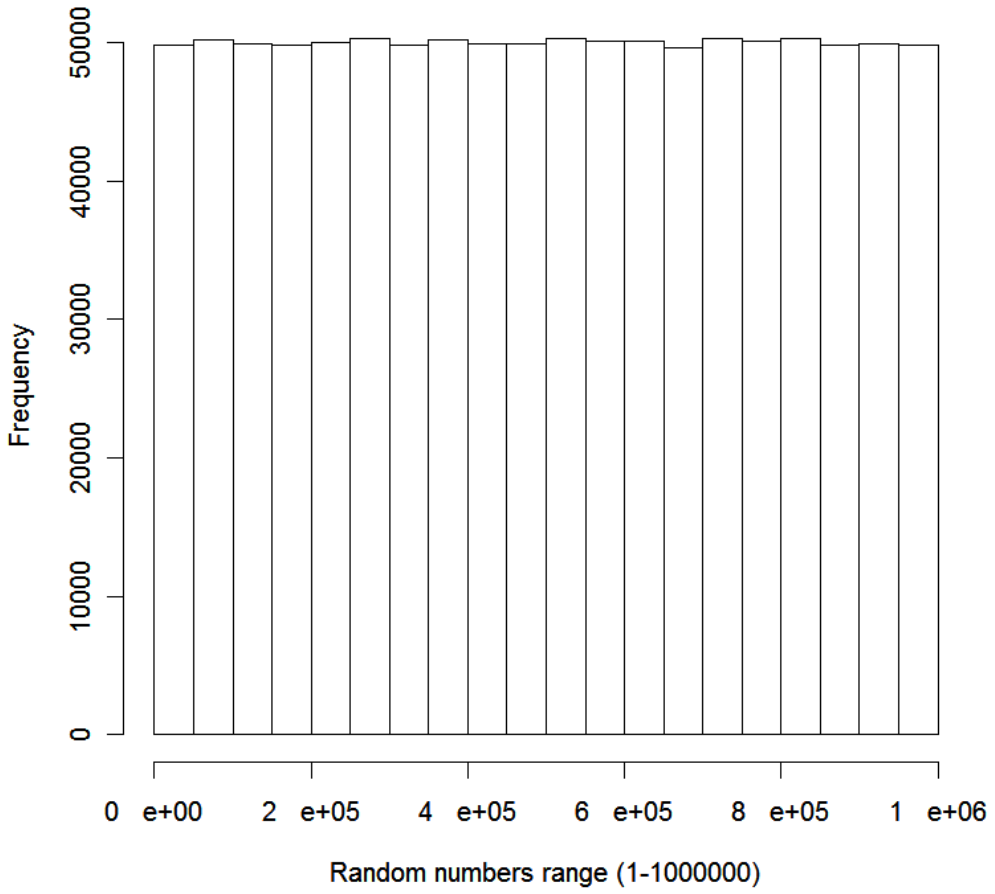

Supplemental figure 1. Uniform distribution created by the random number generator
Uniform distribution of random numbers is shown for the whole range (0-999,999) using a histogram and a bin size of 50,000.

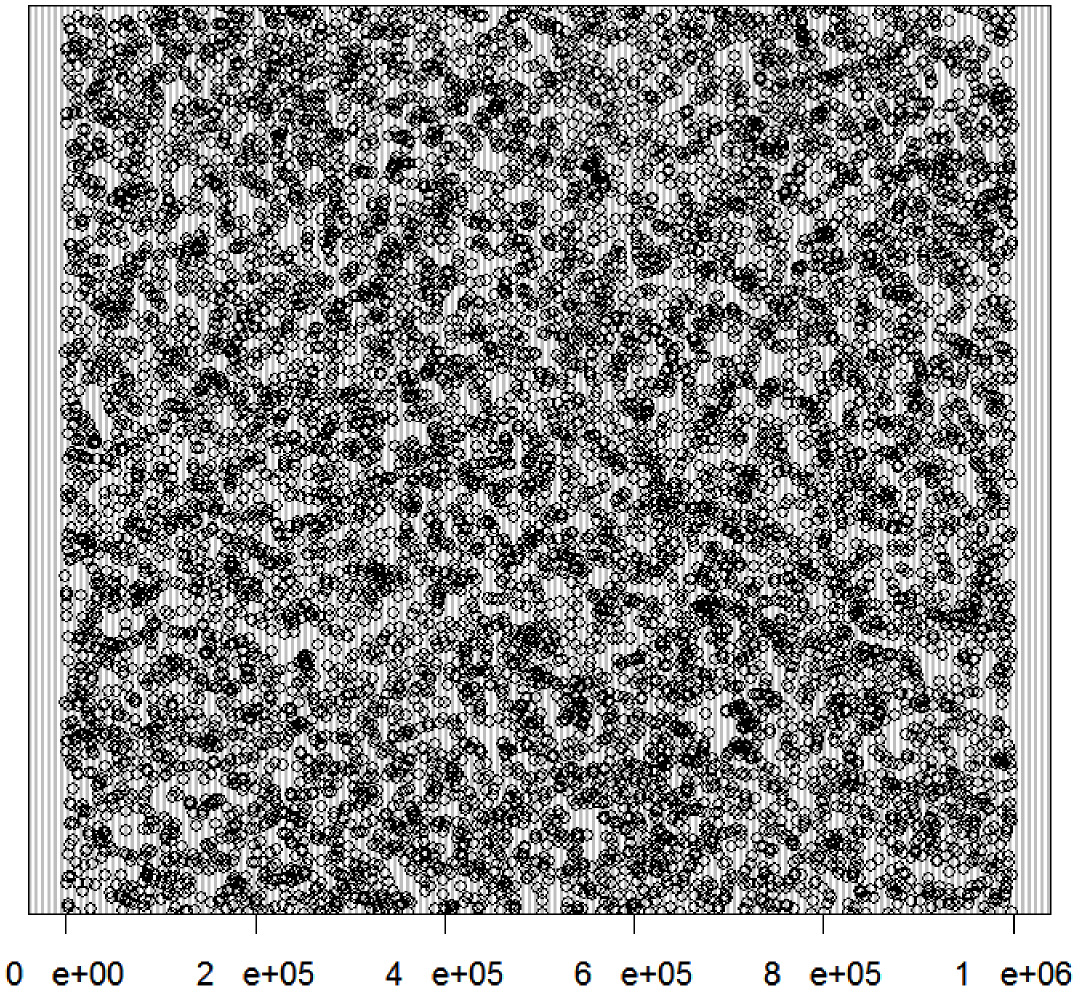

Supplemental figure 2. Uniform distribution created by the random number generator
Uniform distribution of random numbers is shown for the whole range (0-999,999) using a dot-chart.

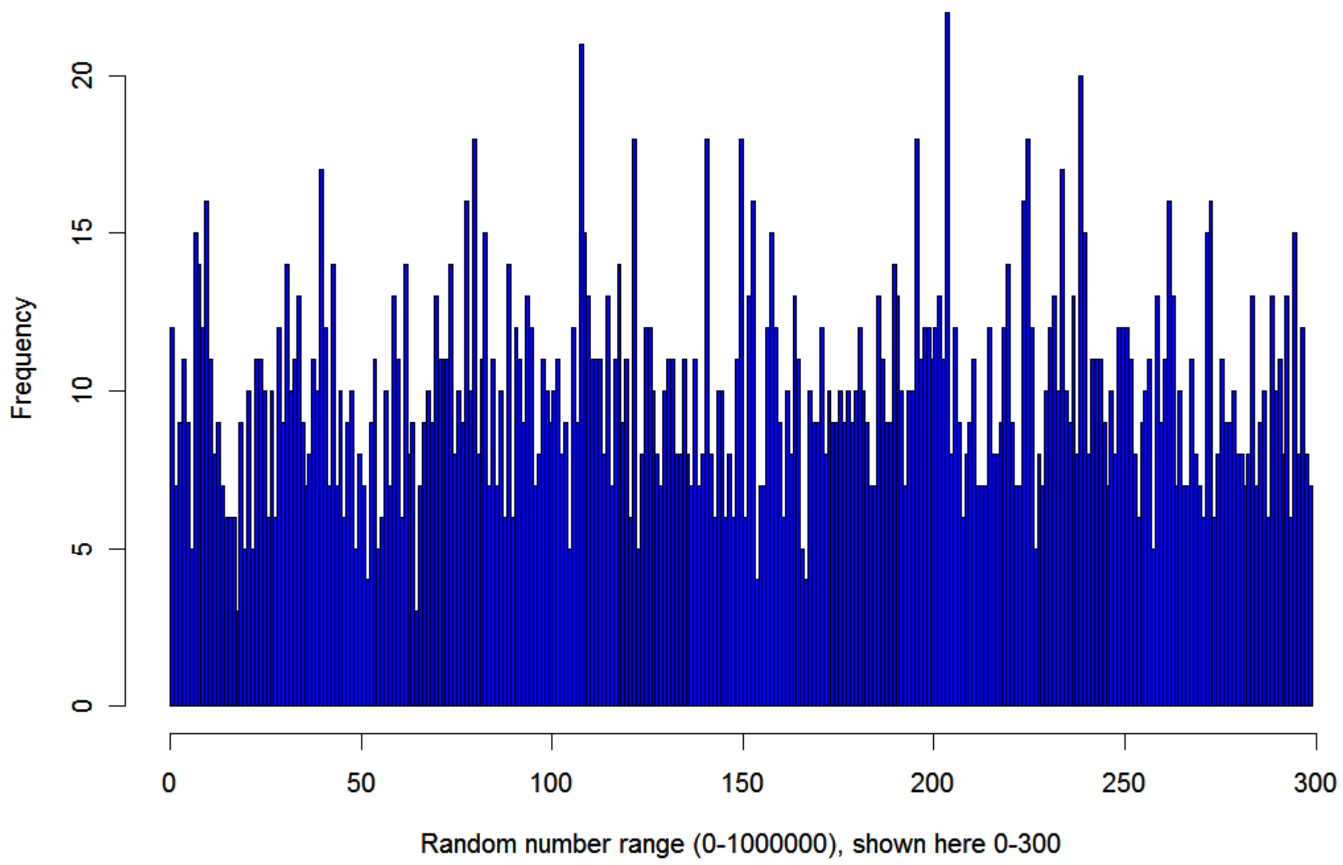

Supplemental figure 3. Uniform distribution created by the random number generator shown at the smaller scale
The distribution of 10,000,000 random numbers from 0-999,999 plotted for the range 0-300, using the bin size of 1.

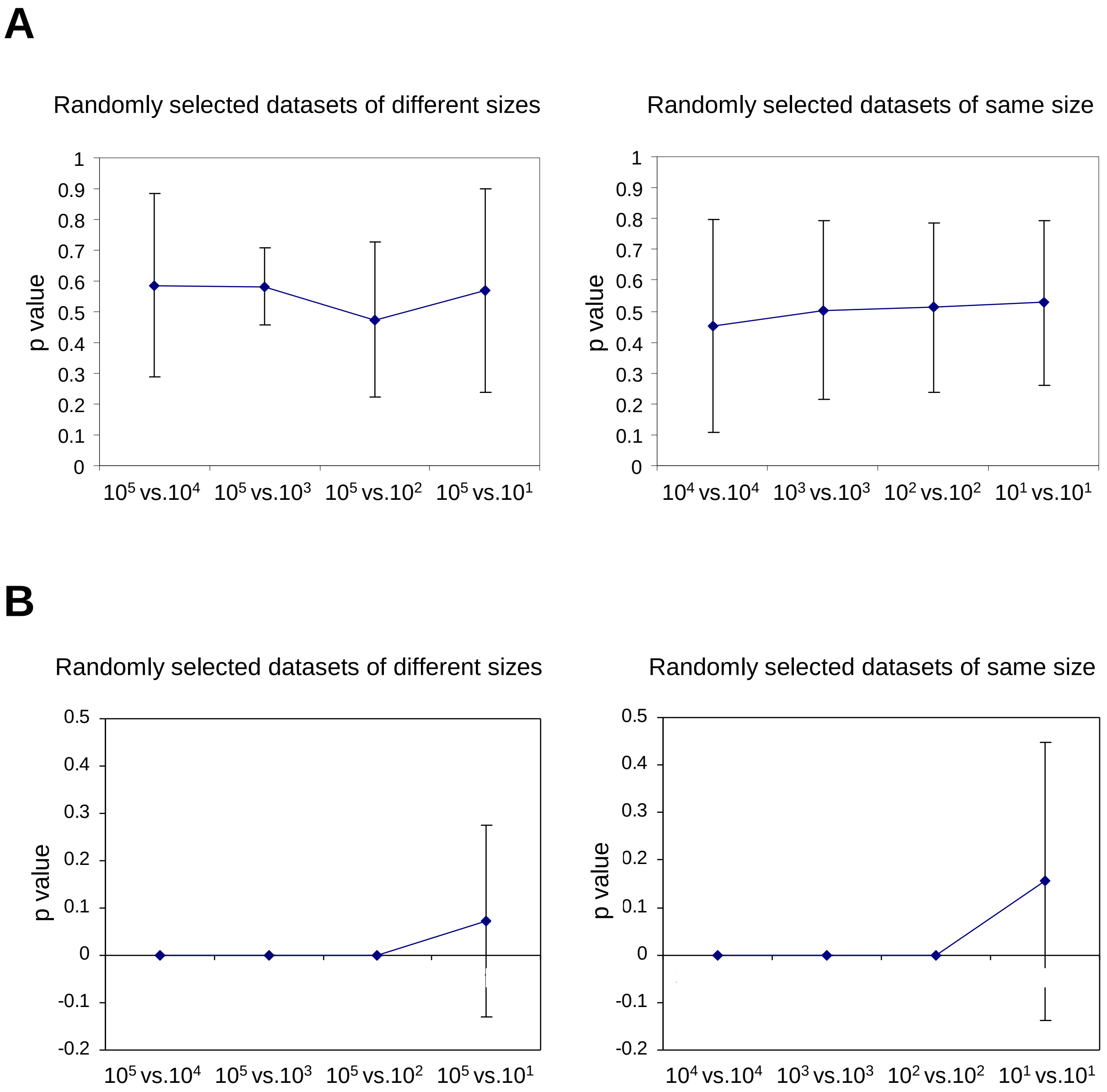

Supplemental figure 4. Statistical properties of random sampling method

Two sets of 10^5 numbers were chosen to have a normal distribution and either identical means of 10, or different means of 10 and 11. The standard deviations were chosen to be the same and equal to 1. We next randomly sub-sampled these datasets to define subsets of either 10^4, 10^3, 10^2 or 10^1 numbers. We performed statistical comparisons of the two datasets using two-tailed t-test either by comparing sampled datasets of the same size or by comparing the sampled sets with the dataset of original size. Each graph shows a distribution of the obtained p-values. **A**. Comparison of samples of equal means. **B**. Comparison of samples of non-equal means.
